# Characterising the allergic profile of children with cystic fibrosis

**DOI:** 10.1002/iid3.540

**Published:** 2021-09-27

**Authors:** Amy L. Faulkner, Michael Grayling, Benjamin Shillitoe, Malcolm Brodlie, Louise J. Michaelis

**Affiliations:** ^1^ Faculty of Medical Sciences Newcastle University Newcastle upon Tyne UK; ^2^ Population Health Sciences Institute, Faculty of Medical Sciences Newcastle University Newcastle upon Tyne UK; ^3^ Department of Immunology, Allergy, and Infectious Diseases, Great North Children's Hospital Newcastle upon Tyne Hospitals NHS Foundation Trust Newcastle upon Tyne UK; ^4^ Translational and Clinical Research Institute, Faculty of Medical Sciences Newcastle University Newcastle upon Tyne UK; ^5^ Paediatric Respiratory Medicine, Great North Children's Hospital Newcastle upon Tyne Hospitals NHS Foundation Trust Newcastle upon Tyne UK

**Keywords:** allergic rhinitis, *Aspergillus*, cystic fibrosis, hypersensitivity

## Abstract

**Background:**

Cystic fibrosis (CF) is a genetic condition that affects multiple organ systems. Allergic bronchopulmonary aspergillosis (ABPA) is a well‐recognised problem but other allergic conditions are less well documented in CF.

**Objective:**

To characterise the allergic profile of a cohort of children with CF, with a focus on those with ABPA.

**Methods:**

A cohort of children with CF were interviewed and retrospective data were collected regarding their allergic histories and other relevant clinical features.

**Results:**

The cohort included 37 children with median age of 9 years (interquartile range: 6‐12). There was a history of ≥1 allergic condition(s) in 28/37 children (76%). The most common allergic condition was allergic rhinitis (AR) in 21/37 (57%) and 16 of these 21 children (76%) had another allergic condition. All children with ABPA (8) had another allergic condition. In some children ABPA exacerbations appeared to be seasonal, suggesting possible cross‐sensitisation between *Aspergillus fumigatus* and aeroallergens associated with seasonal AR. Allergic conditions were also common in children with *Pseudomonas aeruginosa* infection.

## Graphical abstract

“Example allergic timelines for children with CF”

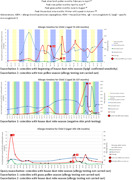

## 1 INTRODUCTION

Cystic fibrosis (CF) is an autosomal recessive genetic condition with a prevalence of around 1:2000 in the United Kingdom (UK).^1^ CF affects many organs but most notably causes problems in the respiratory system, with disordered airway surface liquid homeostasis occurring as a result of defective CF transmembrane conductance regulator function.^2^ This causes impaired mucus clearance, susceptibility to infection and neutrophilic inflammation.^1^, ^3^ *Pseudomonas aeruginosa* and *Aspergillus fumigatus* are amongst key pathogens that drive lung pathology.^3^, ^4^, ^5^

Allergic bronchopulmonary aspergillosis (ABPA) is an allergic condition, which is rare in the general population, but has a prevalence of around 9% in people with CF.^6^ ABPA results from sensitisation to *A. fumigatus* and is characterised by peripheral eosinophilia, elevated serum immunoglobulin E (IgE), episodic bronchial obstruction and lung infiltrates with or without bronchiectasis.^5^ Severe episodes of ABPA can be life‐threatening and require admission to intensive care.^5^ The reasons for the association between CF and ABPA are not well understood.

The pathogenesis of allergic disease is recognised to be multifactorial. According to several well accepted hypotheses, certain allergic conditions are on causative pathways for others, meaning that they tend to accumulate.^7^, ^8^, ^9^, ^10^ The classical order of allergy progression during childhood is known as the “Atopic March”,^11^, ^12^, ^13^ and follows the sequence of: eczema, food allergy, allergic rhinitis (AR) and asthma. Estimated prevalence of these conditions in children in the United Kingdom, as self‐reported in population level data, are eczema 11%–20%, food allergy 8%, AR 10%–15% and asthma 10%.^14^, ^15^, ^16^, ^17^

It is known that ABPA and drug allergy are common in CF,^18^ but besides this, literature regarding the profile of allergy in CF and possible mechanisms involved is limited. One area that has been researched is the role of the gut microbiome in the pathogenesis of ABPA. It is thought that gut dysbiosis (which is common in CF),^19^, ^20^ might lead to dysregulation of the mycobiome, expansion of *A. fumigatus* and predispose individuals to airway sensitisation.^21^ Other possible factors include prolonged exposure to antibiotics, and a susceptibility to lung infection with *P. aeruginosa*, which are both associated with the development of allergy.^22^, ^23^

We undertook a hypothesis‐generating single centre pilot study with the aim of increasing understanding of the allergic profile of children with CF and to explore factors that may contribute to the development of ABPA.

## 2 METHODS

### 2.1 Study design

A retrospective pilot study examining the allergic profile of a cohort of children with CF was carried out between February and July 2020. Participants were interviewed as part of routine clinic review. The sole inclusion criterion was that they had a clear diagnosis of CF.

### 2.2 Data collection

A modified version of the validated “International Study of Asthma and Allergies in Childhood” questionnaire^24^ was used to collect data regarding participants' allergy‐focused history and associated factors (Appendix 2). Results of allergic, immunological and nutritional investigations were collected from participants’ electronic medical records. Where data from the date of interview was not available the most recent results were recorded.

### 2.3 Data and statistical analysis

Analyses were largely descriptive regarding patterns of allergy in the sample population. Allergic outcomes were defined as presence versus absence of allergic conditions.

Summary statistics were computed using the Statistical Package for the Social Sciences (SPSS).^25^ Comparisons of proportions were performed in R,^26^ using the Berger and Boos (1994) method via the Exact package.^27^ Comparison of percentage predicted forced expiratory volume in 1 s (%FEV1) was performed using the Mann–Whitney *U* test in R.^26^ In relation to possible associations between *P. aeruginosa* and allergy and median age at interview the Kruskal–Wallis and Fischer's exact statistical tests were performed using SPSS.^25^ A *p* value <.05 was considered statistically significant throughout. All data were handled in accordance with the General Data Protection Regulations.

## 3 RESULTS

### 3.1 Sample population demographics

In total, 37 participants were sampled as they attended outpatient clinics, (from the GNCH CF clinic of 176 children), with a median age of 9 years (interquartile range: 6–12). Other key demographics of the population and study cohort are provided in Table 1.

**Table 1:** Demographics of the study cohort

					CFTR Genotype			%FEV1
		Age in years Median (IQR)	Male gender	White ethnicity	F508del/F508del	F508del/Other	Other/Other	Median (IQR)
Sample population [*n* = 176]		8.0 (5.0–12.0)^a^	92 (52.3%)	158 (89.8%)	86 (48.9%)	75 (42.6%)	15 (8.5%)	93.7 (83.4–103.8) [*n* = 110] *At most recent annual review*
Study cohort [*n* = 37]	Study cohort [*n* = 37]	9.0 (6.0–12.0)	22 (59.5%)	37 (100%)	23 (62.2%)	13 (35.1%)	1 (2.7%)	95.5 (85.2–103.5) [*n* = 33]
	Eczema [*n* = 18]	8.5 (6.0–11.0)	12 (66.7%)	18 (100%)	11 (61.1%)	6 (33.3%)	1 (5.6%)	95.5 (78.2–98.8) [*n* = 15]
	Seasonal Allergic Rhinitis [*n* = 13]	11.0 (10.0–13.0)	7 (53.8%)	13 (100%)	9 (69.2%)	3 (23.1%)	1 (7.7%)	85.2 (74.3–98.5) [*n* = 13]
	Allergic Bronchopulmonary Aspergillosis [*n* = 8]	14.5 (11.8–16.0)	3 (37.5%)	8 (100.0%)	5 (62.5%)	2 (25.0%)	1 (12.5%)	81.4 (75.9–86.7)
	Perennial Allergic Rhinitis [*n* = 6]	8.0 (6.3–9.0)	4 (66.7%)	6 (100%)	3 (50.0%)	3 (50.0%)	0 (0.0%)	102.3 (91.1–111.4) [*n* = 6]
	Perennial Allergic Rhinitis with seasonal exacerbation [*n* = 2]	13.5 (12.3–14.8)	1 (50.0%)	2 (100%)	1 (50.0%)	1 (50.0%)	0 (0.0%)	86.0 (81.0–91.0) [*n* = 2]
	Drug allergy [*n* = 7]	15.0 (12.0–16.0)	3 (42.9%)	7 (100%)	6 (85.7%)	1 (14.3%)	0 (0.0%)	86.2 (63.5–91.8) [*n* = 7]
	Food allergy [*n* = 6]	9.0 (4.0–11.0)	4 (66.7%)	6 (100%)	2 (33.3%)	4 (66.7%)	0 (0.0%)	95.8 (94.0–97.1) [*n* = 4]
	Asthma [*n* = 3]	11.0 (9.0–11.5)	2 (66.7%)	3 (100%)	1 (33.3%)	2 (66.7%)	0 (0.0%)	96.0 (90.6–102.7) [*n* = 3]
	No history of allergic conditions [*n* = 9]	8.0 (5.0–9.0)	5 (55.6%)	9 (100%)	6 (66.7%)	3 (33.3%)	0 (0.0%)	104.2 (98.0–105.9) [*n* = 8]

Abbreviations: CFTR, cystic fibrosis transmembrane conductance regulator; %FEV1, percentage of predicted forced expiratory volume in 1 s.

^a^ Age as of 08/03/2021.

### 3.2 Allergic profile of the cohort

In the cohort, 28/37 (75.7%) children reported history of ≥1 of the five main allergic conditions. Allergic comorbidity (≥2 of the main allergic conditions), was present in around half of the cohort, including all children with food allergy or asthma. Eight children had ≥3 allergic conditions (all including eczema). Furthermore, 5/6 with food allergy had a history of eczema. All with asthma had AR. None had all five allergic conditions.

**Figure 1:**
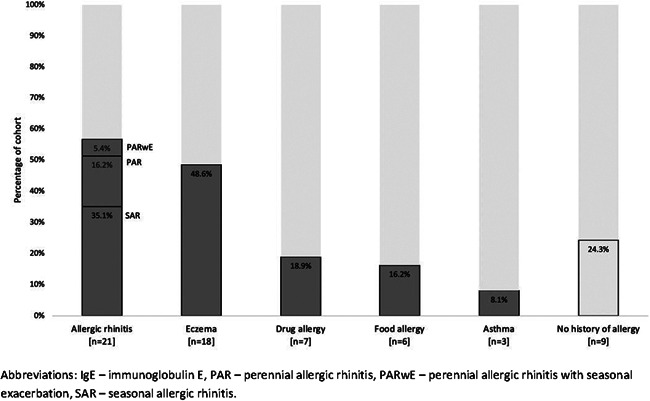
Distribution of the main five allergic conditions in the study cohort. IgE, immunoglobulin E; PAR, perennial allergic rhinitis; PARwe, perennial allergic rhinitis with seasonal exacerbation; SAR, seasonal allergic rhinitis

AR was the most commonly reported condition (Figure 1). All children with AR who had specific IgE testing had sensitivity to >1 aeroallergen. This included half being sensitised to *A. fumigatus*. One child had a history of anaphylaxis (related to food allergy) and two had experienced allergy‐associated breathing difficulties (related to drug allergy). Of those with eczema or AR 88% used daily treatment regimens during symptomatic periods—91% with AR used oral antihistamines, 29% with AR used corticosteroid nasal sprays and two‐thirds with eczema used topical steroid creams. None with eczema, asthma or AR used oral corticosteroids or other immunosuppressants.

Median %FEV_1_ varied between allergic conditions. Those with seasonal allergic rhinitis (SAR) and perennial allergic rhinitis with seasonal exacerbation (PARwE) had the worst lung function and those with perennial allergic rhinitis (PAR) had the best (Table 1).

In terms of median age at time of interview, children with each of the allergic conditions, in ascending order, was: eczema 110 months (*n* = 18), food allergy 113 months (*n* = 21), AR 133 months (*n* = 21), asthma 138 months (*n* = 3) and drug allergy 184 months (*n* = 7). The median ages of each subgroup were significantly different according to the Kruskal–Wallis test (*p* = .004).

### 3.3 CF‐specific features of the cohort

Based on the existing literature, three prominent features of CF were identified as being of interest in terms of possible association with atopic disease:

(a) Prolonged exposure to antibiotics: all were on daily flucloxacillin prophylaxis.
(b) Upper airway pathology: 16/21 (76.2%) of those with AR had allergic comorbidity. Upper airway pathology was also frequent in the ABPA subgroup: half had SAR, 12.5% had PAR and 12.5% had PARwE (Table 2).
(c) Respiratory infection with *P. aeruginosa*: present in 17/37 (49.4%) were and 14/17 of these children had allergy compared to 12/20 who were not infected. The difference in prevalence of AR between those with *P. aeruginosa* and those not (13/17 vs. 8/20) was statistically significant according to the Chi‐square test (*p* = .04).

**Table 2:** Demographics and allergic profile of the ABPA‐positive versus ABPA‐negative subgroups in the study cohort

	Whole cohort	ABPA subgroup	Non‐ABPA subgroup	*p* value comparing subgroups
	*n* = 37 (%)	*n* = 8 (%)	*n* = 29 (%)	
Demographics				
Age in years	9.0 (6.0–12.0)	14.5 (11.8–16.0)	8.0 (5.0–11.0)	*p* = .0005^a^
Gender male	22 (59.5)	3 (8.1)	19 (51.4)	*p* = .140^b^
Reported allergy	28 (75.7)	8 (21.6)	20 (54.1)	*p* = .084^b^
Allergic rhinitis (any)	21 (56.8)	6 (16.2)	15 (40.5)	*p* = .288^b^
Seasonal allergic rhinitis	13 (35.1)	4 (10.8)	9 (24.3)	*p* = .271^b^
Perennial allergic rhinitis	6 (16.2)	1 (2.7)	5 (13.5)	*p* = .936^b^
Perennial allergic rhinitis	2 (5.4)	1 (2.7)	1 (2.7)	*p* = .223^b^
Drug allergy	7 (18.9)	4 (10.8)	3 (8.1)	*p* = .014^b^
Eczema	18 (48.6)	2 (5.4)	16 (43.2)	*p* = .166^b^
Food allergy	6 (16.2)	0 (0.0)	6 (16.2)	*p* = .263^b^
Atopic asthma	3 (8.1)	1 (2.7)	2 (5.4)	*p* = .376^b^
Results				
%FEV1 (at time of interview)	95.8 (84.4–103.8) [*n* = 34]	77.5 (75.7–85.7) [*n* = 7]	97.7 (89.6–104.8) [*n* = 27]	*p* = .032^a^
Specific IgE (KAu/L)				
*Aspergillus fumigatus*	12 (42.9) [*n* = 28]	8 (28.6) [*n* = 8]	4 (14.3) [*n* = 20]	*p* = .00005^b^
Grass Mix 1	4 (80.0) [*n* = 5]	2 (80.0) [*n* = 3]	2 (40.0) [*n* = 2]	*p* = .568^b^
House Dust Mite	2 (66.7) [*n* = 3]	1 (66.7) [*n* = 2]	1 (33.3) [*n* = 1]	*p* = .750^b^
Tree Mix 1	0 (0.0) [*n* = 4]	0 (0.0) [*n* = 3]	0 (0.0) [*n* = 1]	*p* = 1.000^b^
Weed Mix 1	0 (0.0) [*n* = 3]	0 (0.0) [*n* = 2]	0 (0.0) [*n* = 1]	*p* = 1.000^b^
Cat dander	2 (66.7) [*n* = 3]	0 (0.0) [*n* = 1]	2 (66.7) [*n* = 2]	*p* = .250^b^
Dog dander	1 (50.0) [*n* = 2]	0 (0.0) [*n* = 1]	1 (50.0) [*n* = 1]	*p* = .500^b^
Moulds Mix	1 (100.0) [*n* = 1]	1 (100.0) [*n* = 1]	0 (0.0) [*n* = 0]	*P* = N/A
Risk factors				
Prolonged antibiotic exposure	37 (100.0)	8 (21.6%)	29 (78.4)	*p* = 1.000^b^
Chronic infection with *Pseudomonas aeruginosa*	17 (45.9)	6 (16.2)	11 (29.8)	*p* = .082^b^

Abbreviations: ABPA, allergic bronchopulmonary aspergillosis; %FEV1, percentage of predicted forced expiratory volume in 1 s; IQR, interquartile range; SpIgE, specific immunoglobulin E.

^a^ Statistical test used: Mann–Whitney *U*.

^b^ Statistical test used: Berger and Boos. A *p* value of <.05 considered statistically significant.

### 3.4 Analysis of children with ABPA

Overall, 8/37 (21.6%) of the cohort had ABPA (diagnostic criteria in Appendix 1) and the median age of this subgroup was 9 years (Table 2). Median %FEV1 was 20.2% points lower in those with ABPA compared to those without. Other co‐existent allergic conditions included AR (75%) and drug allergy (50%). The difference in prevalence of drug allergy between the whole cohort and the ABPA subgroup was statistically significant according to the Berger and Boos test (*p* = .014).

Having been identified as a subgroup of interest, retrospective allergic timelines for six children with ABPA from the cohort were compiled (Figure 2). These comprised serial total IgE (TIgE), eosinophil, basophil and lymphocyte counts and dates of hospital admissions due to ABPA exacerbations. Timeline length depended on availability of data and spanned between 6 and 10 years per child.

Figure 2Allergic timelines for six children with allergic bronchopulmonary aspergillosis in the study cohort. In the United Kingdom peak months for silver birch pollen are February to April, tree pollen April to June, grass pollen June to August and house dust mite in Autumn and Winter. ABPA, allergic bronchopulmonary aspergillosis; HDM, house dust mite; IgE, immunoglobulin E, SpIgE, specific immunoglobulin E.

**Figure:**
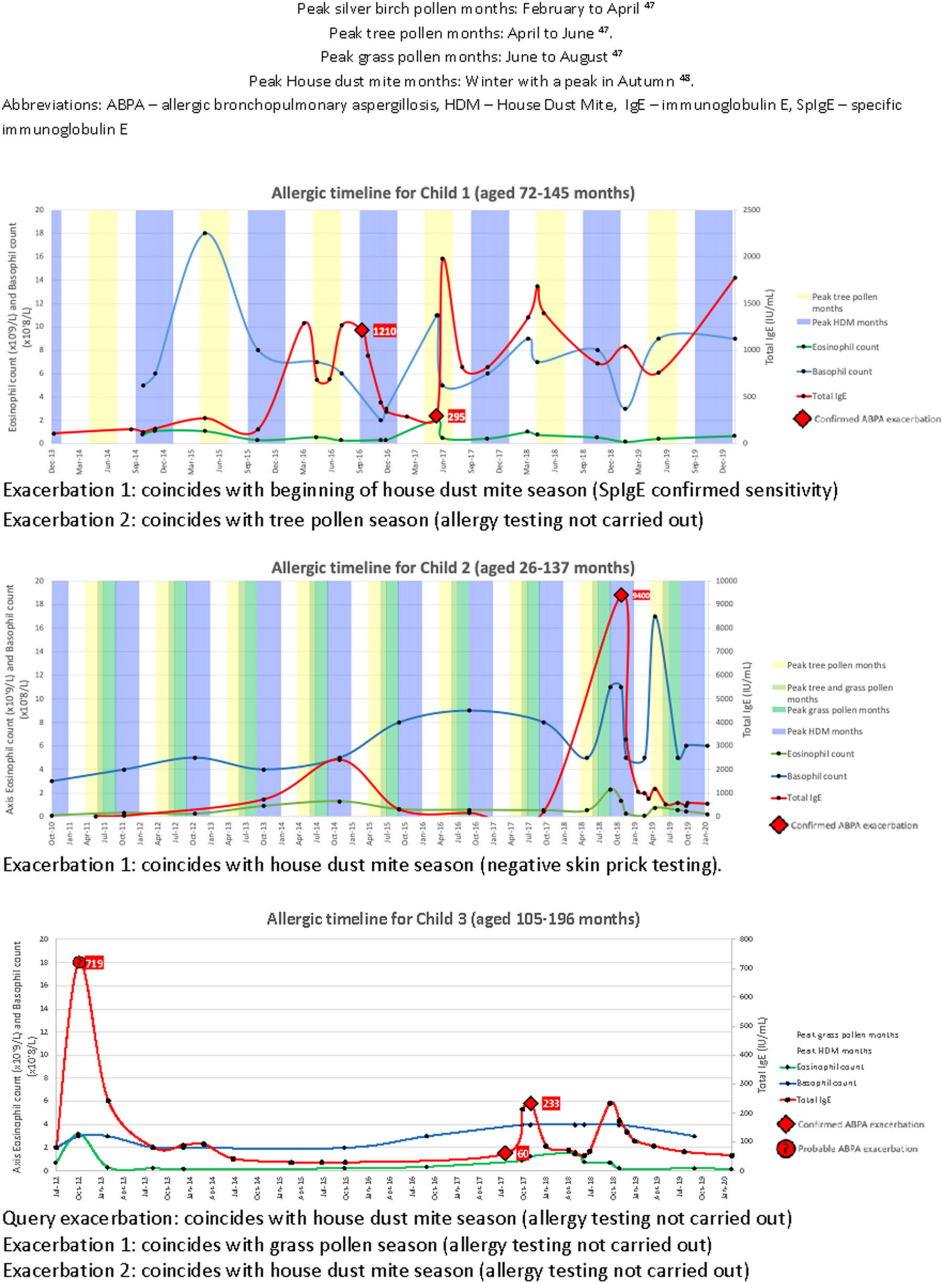

**Figure:**
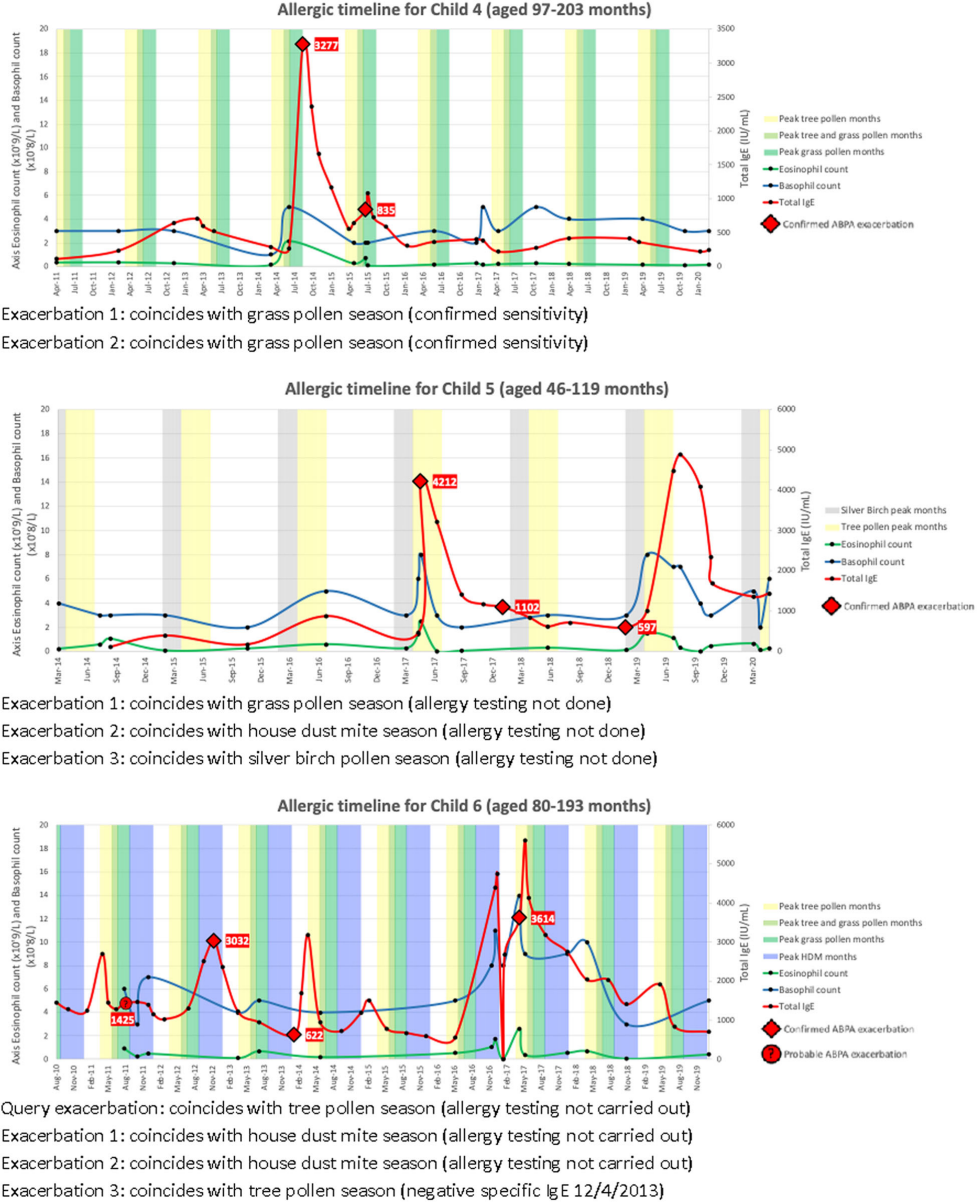

TIgE was raised during all ABPA exacerbations. Eosinophil, basophil and lymphocyte counts were measured during hospital admission for eight exacerbations. Eosinophil count was above normal in 7/8 exacerbations and basophil count in 6/8 (normal ranges in Appendix 1). ABPA exacerbations sometimes coincided with the peak months of certain aeroallergens (Figure 2).

## 4 DISCUSSION

This pragmatic study of the allergic profile of a cohort of children with CF has produced several interesting but preliminary findings. Firstly, the prevalence of allergic conditions was more common in this cohort than has been estimated in the general UK paediatric population (albeit figures can not be directly compared due to differences in methodology).^14^, ^15^, ^16^, ^17^, ^28^ The second observation is that children with ABPA often have other allergic conditions.

There are several features in children with CF that may make allergic conditions more likely. Firstly, gut dysbiosis (which has long been implicated in allergy)^29^, ^30^, ^31^, ^32^ may contribute to ABPA pathogenesis via the gut‐lung axis.^21^ Furthermore, chronic infection and prolonged antibiotic exposure have been linked with allergy through T_h_2 skewing, and both were prolific in the cohort.^22^ Antibiotic use has also been identified as a factor for drug allergy,^33^, ^34^, ^35^ and it is probable that the widespread use in this cohort contributed to the frequency of drug reactions seen here.

Chronic respiratory infection with *P. aeruginosa* appeared to be associated with allergy. This is in accordance with recent literature suggesting that *P. aeruginosa* infection might contribute to allergy by stimulating histamine production^23^ in addition to resulting in increased antibiotic exposure.^22^ There is also evidence to suggest that *P. aeruginosa* and *A. fumigatus* interact in the airway, though opinion is divided as to whether the relationship is mutually beneficial or antagonistic.^36^ If mutually beneficial then *P. aeruginosa* may facilitate *A. fumigatus* to persist in the airways and predispose individuals to become sensitised to the fungus, resulting in ABPA.

AR was frequent in this cohort of children. Children with CF have defective mucosal clearance which may permit aeroallergens to persist in the upper airway. In combination with inflammatory changes to the airway epithelium this may create an environment highly conducive to sensitisation.^3^ This is analogous to concepts described in the “Dual Exposure” hypothesis,^7^, ^8^, ^9^ though in this context sensitisation might occur at damaged epithelia in the airway rather than through damaged skin. Sensitisation to *A. fumigatus* was observed in ABPA and AR, suggesting a possible role for the fungus in producing symptoms in both the upper and lower airways. AR was seen to precede ABPA, which if reproducible on a larger scale, might suggest that *A. fumigatus‐*associated allergy first arises in the upper airway in the form of AR before contributing to the pathogenesis of lower airway allergy (ABPA). This is in accordance with the “United Airways” hypothesis.^10^ Furthermore, some with ABPA had apparent seasonality of exacerbations suggestive of a possible interaction between aeroallergens classically associated with SAR and ABPA. This concept is not previously described in the literature, but is comparable to the relationship between AR and atopic asthma observed in phenomena such as “thunderstorm asthma”.^10^, ^37^, ^38^

Comparing the median ages of each allergic subgroup in the study cohort provides some suggestions as to the chronology of allergy in CF, which appears similar to that in the general population^11^, ^12^, ^13^ but with additional features (chronic *P. aeruginosa* infection, ABPA and drug allergy). It is important to note that this was based on age at time of interview as age of onset of the conditions was not elicited.

It may seem counter‐intuitive that reported asthma was uncommon in what appears to be a highly allergic group of children. However, respiratory symptoms are common in children with CF and there is often overlap, making it challenging to distinguish between CF‐related symptoms and asthma.

### 4.1 Limitations

Limitations to this pragmatic study include the restricted cohort size, possible recruitment bias and missing data. The COVID‐19 pandemic limited the scale of the study. However, the demographics of the study cohort are broadly similar to the whole clinic and these preliminary observations are useful to trigger further research.

### 4.2 Future directions

No firm conclusions can be drawn about the allergic profile of children with CF from a retrospective study of this size and nature, although it has produced findings that should prompt further research to characterise the allergic profile of children with CF. This would involve multi‐centre allergy testing and prospective monitoring of allergic disease and risk factors of children with CF. We propose that further understanding of Th2 allergic inflammation in children with CF is required to elucidate the immune mechanisms involved and to potentially facilitate the use of targeted therapies to improve quality of life in this chronic disease.

## CONFLICT OF INTERESTS

Malcolm Brodlie reports investigator‐led research grants from Pfizer and Roche Diagnostics; speaker fees paid to Newcastle University from Novartis, TEVA and Roche Diagnostics and travel expenses to educational meetings from Boehringer Ingelheim and Vertex Pharmaceuticals. Louise J. Michaelis reports investigator‐led research grants from Danone and Sanofi; Speaker fees paid to Newcastle University from Danone and Sanofi, advisory board and travel expenses to educational meetings from Danone, Novartis, Allergy Therapeutics and Sanofi Pharmaceuticals. The other authors have no conflict of interests.

## AUTHOR CONTRIBUTIONS

**Amy L. Faulkner**: investigation, methodology, formal analysis, writing—original draft. **Michael Grayling**: formal analysis, writing—review and editing. **Benjamin Shillitoe**: formal analysis, writing—review and editing. **Malcolm Brodlie**: conceptualization, methodology, formal analysis, supervision, writing—review and editing. **Louise J. Michaelis**: conceptualization, methodology, formal analysis, supervision, writing—review and editing.

## ETHICAL STATEMENT

The research was registered (audit reference 10146) and initiated as part of a service evaluation of allergy care provided to children with CF at the Great North Children's Hospital (GNCH) in accordance with national clinical guidelines, both with regards to CF and allergy^39^, ^40^, ^41^, ^42^, ^43^, ^44^

## Supporting information

Supporting information.
