# Supplementary material for: Characterising the allergic profile of children with cystic fibrosis
Source: Immun Inflamm Dis. 2021 Sep 27;10(1):60–9. doi: 10.1002/iid3.540 (PMC8669696; doi:10.1002/iid3.540)
Supplement: Supplementary file 1 — Supporting information. [file IID3-10-60-s001.docx]

# Appendices

## Appendix 1: Definitions and diagnostic criteria

| Allergic Conditions | |
| --- | --- |
| Allergic Bronchopulmonary Aspergillosis^1^ | Isolation of *Aspergillus fumigatus* from a respiratory sample or raised IgE in combination with   1. Reduced lung function 2. Respiratory symptoms in the form of wheeze 3. Shortness of breath and/or cough. 4. Symptomatic treatment with oral prednisolone +/- antifungals. |
| Allergic Rhinitis Perennial Allergic Rhinitis (PAR)  Seasonal Allergic Rhinitis (SAR)  Perennial Allergic Rhinitis with seasonal exacerbation (PARwe) | Participant positive for condition if:   1. It had been diagnosed by medical professional   OR   1. They had self-reported symptoms consistent with the condition as per the ISAAC questionnaire^2^, e.g. answered yes when asked: 2. “*Have you ever had a problem with sneezing, or a runny, or blocked nose when you DID NOT have a cold or the flu"*   PAR: symptoms every month of the year.  SAR: symptoms only in some months of the year.  PARwE: symptoms every month of the year but worse in certain months. |
| Atopic Asthma | Participant positive for condition if:   1. It had been diagnosed by medical professional   OR   1. They had self-reported symptoms consistent with the condition as per the ISAAC questionnaire^2^, e.g. answered yes when asked: 2. *“Has your child ever had wheezing or whistling in the chest at any time in the past?”* |
| Drug Allergy | Participant positive for condition if:   1. It had been diagnosed by medical professional   OR   1. They had self-reported symptoms consistent with the condition as per the RCPCH care pathway^3^, e.g. answered yes when asked: 2. *“Has your child ever experienced an allergic reaction to a prescribed drug?”* |
| Eczema | Participant positive for condition if:   1. It had been diagnosed by medical professional   OR   1. They had self-reported symptoms consistent with the condition as per the ISAAC questionnaire^2^, e.g. answered yes when asked: 2. *“Has your child ever had an itchy rash which was coming and going for at least six months?”* |
| Food Allergy | Participant positive for condition if:   1. It had been diagnosed by medical professional   OR   1. They had self-reported symptoms consistent with the condition as per the RCPCH care pathway^4^, e.g. answered yes when asked: 2. *“Has your child ever had an allergic reaction to something they have eaten?”* |

| Other definitions | |
| --- | --- |
| Aeroallergens included in allergy testing | House dust mite, moulds mix, grass mix, tree mix, weed mix, silver birch, dog, cat. |
| Basophil Count | Normal range as per the Newcastle upon Tyne Hospitals NHS Foundation Trust^5^: 0.00 - 0.10 x 10'9/L |
| Cystic Fibrosis | Children with a clear diagnosis of cystic fibrosis under the care of the Great North Children’s Hospital’s regional multi-disciplinary team. |
| Eosinophil Count | Age-specific normal ranges as per the Newcastle upon Tyne Hospitals NHS Foundation Trust^5^: between 0.3-0.8 and 0.04-0.4 x 10^9^/L |
| Total Immunoglobulin E | Normal ranges as per the Newcastle upon Tyne Hospitals NHS Foundation Trust^5^:  Neonates 0-1.5 IU/L), infants 0-15 IU/L, children aged 1-5 years 0-60 IU/L, children aged 6-9 years 0-90 IU/L, children aged 10-15 years 0-200 IU/L, 16 years and over 0-100 IU/L. |

^1^Sisodia J, Bajaj T. Allergic Bronchopulmonary Aspergillosis. [Updated 2021 Mar 2]. In: StatPearls [Internet]. Treasure Island (FL): StatPearls Publishing; 2021 Jan-. Available from: <https://www.ncbi.nlm.nih.gov/books/NBK542329/>

^2^ The International Study of Asthma and Allergies in Childhood (ISAAC). Worldwide variations in the prevalence of asthma symptoms: the International Study of Asthma and Allergies in Childhood (ISAAC). Eur Respir J. 1998;12(2):315-335

^3^ Royal College of Paediatrics and Child Health, 2011. Allergy care pathway for drug allergy. Available at: <https://www.rcpch.ac.uk/resources/allergy-care-pathway-drug-allergy>. [Accessed 07/04/2021]

^4^ Royal College of Paediatrics and Child Health, 2011. Allergy care pathway for food allergy. Available at: <https://www.rcpch.ac.uk/resources/allergy-care-pathway-food-allergy>. [Accessed 07/04/2021]

^5^ The Newcastle upon Tyne Hospitals Trust. Home - Newcastle Laboratories. <https://www.newcastlelaboratories.com/>. Published 2020. Accessed 14/11/2020, 2020.

## Appendix 2: Modified ISAAC questionnaire used

Questions regarding drug and food allergy, not ordinarily included in the International Study of Asthma and Allergies in Childhood (ISAAC) questionnaire, were additional as per the Royal College of Paediatrics and Child Health Allergy Pathways^1,2^. Appendix 1 outlines the definitions and diagnostic criteria used for conditions examined in the study.

^1^Royal College of Paediatrics and Child Health, 2011. Allergy care pathway for drug allergy. Available at: <https://www.rcpch.ac.uk/resources/allergy-care-pathway-drug-allergy>. [Accessed 07/04/2021]

^2^ Royal College of Paediatrics and Child Health, 2011. Allergy care pathway for food allergy. Available at: <https://www.rcpch.ac.uk/resources/allergy-care-pathway-food-allergy>. [Accessed 07/04/2021]

**FOOD ALLERGY**

1. Has your child ever had an allergic reaction to something they have eaten?

| Yes ☐ | No ☐ | Don’t Know ☐ |
| --- | --- | --- |

*IF THE ANSWER TO a) IS YES, COMPLETE QUESTION. IF NO, SKIP TO NEXT QUESTION*

1. Which food has caused your child their MOST SEVERE allergic reaction? This may have been the ONLY reaction)
2. Thinking about the symptoms of your child’s allergy/allergies please consider the symptoms associated with his or her MOST SEVERE reaction (this may have been the ONLY reaction) and tick all that apply.

| Vomiting ☐ | Abdominal pain ☐ |
| --- | --- |
| Irritability ☐ | Rash ☐ |
| Face Swelling ☐ | Tingling or sore mouth ☐ |
| Swelling of the lips or tongue ☐ | Throat tightening or difficulty swallowing ☐ |
| Breathing difficulties ☐ | Wheeze ☐ |
| Blue around the lips ☐ | Collapse or faint ☐ |
| Other (please specify): | |

1. Thinking about the MOST SEVERE allergic reaction that your child has experienced:
2. What treatment did your child take?

| Anti-Histamines ☐ | Adrenaline Injection (Epi-Pen) ☐ | None ☐ |
| --- | --- | --- |
| Oral Steroid ☐ | Salbutamol Inhaler ☐ | Don’t know ☐ |

1. Did your child go to hospital for the reaction?

| Yes ☐ | No ☐ | Don’t Know ☐ |
| --- | --- | --- |

1. If 4d)ii was yes, did your child stay overnight in hospital?

| Yes ☐ | No ☐ | Don’t Know ☐ |
| --- | --- | --- |

1. Was your child admitted to:

| High Dependency Ward ☐ | Other Ward ☐ | Don’t know ☐ |
| --- | --- | --- |

1. Apart from the most severe reaction, has your child had any allergic reactions to other foods? If so state what he or she had a reaction to.
2. Has your child ever had any of the following?

| Difficulty swallowing ☐ | Pain during swallowing ☐ | Inability to swallow ☐ |
| --- | --- | --- |
| None ☐ | Don’t know ☐ |  |

**NON-FOOD ALLERGY**

1. Has your child ever experienced an allergic reaction to something other than a food or a drug?

| Yes ☐ | No ☐ | Don’t Know ☐ |
| --- | --- | --- |

*IF THE ANSWER TO a) IS YES, COMPLETE QUESTION. IF NO, SKIP TO NEXT QUESTION*

1. Thinking about your child’s MOST SEVERE non-food reaction, what were they allergic to?
2. Thinking about the symptoms of your child’s non-food allergy/allergies please consider the symptoms associated with his or her MOST SEVERE reaction (this may have been the ONLY reaction) and tick all that apply.

| Vomiting ☐ | Abdominal pain ☐ |
| --- | --- |
| Irritability ☐ | Rash ☐ |
| Face Swelling ☐ | Tingling or sore mouth ☐ |
| Swelling of the lips or tongue ☐ | Throat tightening or difficulty swallowing ☐ |
| Breathing difficulties ☐ | Wheeze ☐ |
| Blue around the lips ☐ | Collapse or faint ☐ |
| Other (please specify): | |

1. Thinking about the most severe allergic reaction that your child has experienced:
2. What treatment did your child take?

| Anti-Histamines ☐ | Adrenaline Injection (Epi-Pen) ☐ | None ☐ |
| --- | --- | --- |
| Oral Steroid ☐ | Salbutamol Inhaler ☐ | Don’t know ☐ |
|  |  |  |

1. Did your child go to hospital for the reaction?

| Yes ☐ | No ☐ | Don’t Know ☐ |
| --- | --- | --- |

1. If d)ii. was yes, did your child stay overnight in hospital?

| Yes ☐ | No ☐ | Don’t Know ☐ |
| --- | --- | --- |

1. Was your child admitted to:

| High Dependency Ward ☐ | Other Ward ☐ | Don’t know ☐ |
| --- | --- | --- |

1. Have any of your child’s allergies (food, drug or other) been diagnosed by a medical professional?

| Yes ☐ | No ☐ | Don’t Know ☐ |
| --- | --- | --- |

1. If e) was Yes, which allergy or allergies have been diagnosed?

*IF THE ANSWER TO e) IS YES, COMPLETE QUESTION. IF NO, SKIP TO NEXT QUESTION*

1. Has your child been referred to an allergy specialist? If so, for which allergy?

| Yes ☐ | Allergy: **_______________________** |
| --- | --- |
| No ☐ | Don’t Know ☐ |

1. Does your child have an Adrenaline Auto-injector (EpiPen/JEXT)

| Yes ☐ | No ☐ | Don’t Know ☐ |
| --- | --- | --- |

**DRUG REACTIONS**

1. Has your child ever experienced an allergic reaction to a prescribed drug?

| Yes ☐ | No ☐ | Don’t Know ☐ |
| --- | --- | --- |

*IF THE ANSWER TO a) IS YES, COMPLETE QUESTION. IF NO, SKIP TO NEXT QUESTION*

1. Thinking about your child’s MOST SEVERE drug reaction, which drug did they react to?
2. Thinking about the symptoms of your child’s non-food allergy/allergies please consider the symptoms associated with his or her MOST SEVERE reaction (this may have been the ONLY reaction) and tick all that apply.

| Vomiting ☐ | Abdominal pain ☐ |
| --- | --- |
| Irritability ☐ | Rash ☐ |
| Face Swelling ☐ | Tingling or sore mouth ☐ |
| Swelling of the lips or tongue ☐ | Throat tightening or difficulty swallowing ☐ |
| Breathing difficulties ☐ | Wheeze ☐ |
| Blue around the lips ☐ | Collapse or faint ☐ |
| Headache ☐ | Blood in Stool ☐ |
| Diarrhoea ☐ | Localised reaction to infusion site (If IV drug) ☐ |
| Other (please specify): | |

1. Thinking about the most severe allergic reaction that your child has experienced:
2. What treatment did your child take?

| Anti-Histamines ☐ | Adrenaline Injection (Epi-Pen) ☐ |  |
| --- | --- | --- |
| Oral Steroid ☐ | Salbutamol Inhaler ☐ | None ☐ |
| IV Steroids ☐ | IV Antihistamine ☐ | Don’t know ☐ |
|  |  |  |

1. Did the reaction occur in hospital or elsewhere? (e.g: at home)

| Hospital ☐ | Elsewhere ☐ |
| --- | --- |

1. If the answer to ii. was elsewhere, was your child taken to hospital because of their reaction?

| Yes ☐ | No ☐ |
| --- | --- |

1. If iii. was yes, was your child admitted to:

| High Dependency Ward ☐ | Other Ward ☐ | Don’t know ☐ |
| --- | --- | --- |

1. Apart from the most severe reaction, has your child had any allergic reactions to other prescribed drugs? If so state which drug/drugs he or she has had a reaction to

**ECZEMA**

1. Has your child ever been diagnosed with **Eczema/Atopic Dermatitis**?

| Yes ☐ | No ☐ | Don’t Know ☐ |
| --- | --- | --- |

1. Has your child ever had an itchy rash which was coming and going for at least six months?

| Yes ☐ | No ☐ | Don’t Know ☐ |
| --- | --- | --- |

*IF THE ANSWER TO a) OR b) IS YES, COMPLETE QUESTION. IF NO, SKIP TO NEXT QUESTION*

1. Has your child had this itchy rash at any time within the last 12 months?

| Yes ☐ | No ☐ | Don’t Know ☐ |
| --- | --- | --- |

1. Has this itchy rash affected any of the following places at any time? (i.e. not just within the last 12 months)

*Folds of elbows, behind the knees, in front of ankles, under the buttocks, or around the neck, ears or eyes?*

| Yes ☐ | No ☐ | Don’t Know ☐ |
| --- | --- | --- |

1. At what age did this rash first occur?

| Under 2 years old ☐ | 2-4 years old ☐ | 5 or older ☐ | Don’t know ☐ |
| --- | --- | --- | --- |

1. In the past 12 months, how often on average has your child been kept awake at night by this itchy rash?

| Never in the past 12 months ☐ |
| --- |
| Less than one night per week ☐ |
| One or more nights a week ☐ |

1. Has your child ever been prescribed topical steroid cream for this rash?

| Yes ☐ | No ☐ | Don’t Know ☐ |
| --- | --- | --- |

1. Tick any of the following treatments that your child requires most days for their eczema when it is present:

| Emollients ☐ | Topical Steroid creams ☐ | Soap Substitute ☐ |
| --- | --- | --- |
| Bath Additives ☐ | Oral Steroids ☐ | IV Steroids ☐ |
| Skinny Garments ☐ | Icthopaste Bandages ☐ | Immune Suppressive ☐ |
| Biologics ☐ |  | None ☐ |

**ATOPIC ASTHMA**

1. Has your child been diagnosed with Atopic Asthma?

| Yes ☐ | No ☐ | Don’t Know ☐ |
| --- | --- | --- |

1. Has your child’s chest ever sounded wheezy?

| Yes ☐ | No ☐ | Don’t Know ☐ |
| --- | --- | --- |

*IF THE ANSWER TO a) OR b) IS YES, COMPLETE QUESTION. IF NO, SKIP TO NEXT QUESTION*

1. How many attacks of wheezing has your child had in the last 12 months?

| None ☐ | 1 to 3 ☐ | 4 to 12 ☐ | More than 12 ☐ |
| --- | --- | --- | --- |

1. In the past 12 months, how often has your child’s sleep been disturbed due to wheezing?

| Never woken with wheezing ☐ |
| --- |
| Less than one night per week ☐ |
| One or more nights per week ☐ |
| Don’t know ☐ |
|  |

1. In the past 12 months, has wheezing ever been severe enough to limit your child’s speech to only one or two words at a time between breaths?

| Yes ☐ | No ☐ | Don’t Know ☐ |
| --- | --- | --- |

1. Has your child ever sounded wheezy during or after exercise?

| Yes ☐ | No ☐ | Don’t Know ☐ |
| --- | --- | --- |
|  |  |  |

1. Has your child sounded wheezy during or after exercise in the last 12 months?

| Yes ☐ | No ☐ | Don’t Know ☐ |
| --- | --- | --- |

1. Has your child been prescribed any of the following inhalers for asthma or wheeze?

| Reliever inhaler (blue) ☐ | Preventor inhaler (brown) ☐ |
| --- | --- |
| Neither ☐ | Don’t Know ☐ |

1. Has your child ever been prescribed oral steroids for asthma or wheeze?

| Yes ☐ | No ☐ | Don’t Know ☐ |
| --- | --- | --- |

1. In the last 12 months, has your child had a dry cough at night that wasn’t associated with a cold or a chest infection?

| Yes ☐ | No ☐ | Don’t Know ☐ |
| --- | --- | --- |

**ALLERGIC RHINITIS**

- 1. Has your child been diagnosed with **Hayfever** / **Allergic Rhinitis**?

| Yes ☐ | No ☐ | Don’t Know ☐ |
| --- | --- | --- |

- 1. Does your child ever experience an itchy and/or runny or blocked nose when they do not have a cold?

| Yes ☐ | No ☐ | Don’t Know ☐ |
| --- | --- | --- |

*IF THE ANSWER TO a) OR b) IS YES, COMPLETE QUESTION. IF NO, SKIP TO NEXT QUESTION*

- 1. In which of the past 12 months did this nose problem occur?

| January ☐ | April ☐ | July ☐ | October ☐ |
| --- | --- | --- | --- |
| February ☐ | May ☐ | August ☐ | November ☐ |
| March ☐ | June ☐ | September ☐ | December ☐ |
| None ☐ | Don’t Know ☐ |  |  |

- 1. Does your child require any of the following treatments MOST DAYS during the problematic months?

| Antihistamines ☐ | Inhaled Nasal Steroids ☐ |
| --- | --- |
| Eye Drops ☐ | Anti-Leukotriene Antagonist ☐ |
| Sublingual Immunotherapy ☐ | Subcutaneous Immunotherapy ☐ |
| Oral Steroids ☐ |  |
| None ☐ | Don’t know ☐ |

- 1. In the last 12 months, how much did this nose problem interfere with your child’s daily activities?

| Not at all ☐ | A Little ☐ | A Moderate amount ☐ | A lot ☐ |
| --- | --- | --- | --- |

1. Has your child ever been diagnosed with Allergic Bronchopulmonary Aspergillosis

| Yes ☐ | No ☐ | Don’t Know ☐ |
| --- | --- | --- |

1. If 7a) was yes, has your child received oral steroids for the condition?

| Yes ☐ | No ☐ | Don’t Know ☐ |
| --- | --- | --- |

1. If 7a) was yes, has your child received other medication for the condition apart from oral steroids?

| Yes ☐ | No ☐ | Don’t Know ☐ |
| --- | --- | --- |

**GENERAL MEDICAL HISTORY**

1. Thinking about your child’s current or past feeding (depending on their age)
   1. Does / Did your child have ….

| Breast Milk only ☐ | Formula Milk only ☐ | Breast Milk and Formula milk ☐ |
| --- | --- | --- |

- 1. If your child has / had formula milk, what is the name or brand?

1. Does your child have any other medical conditions for which he/she sees the doctor regularly about? If yes please state condition(s)

| Yes ☐ |
| --- |
| No ☐ |
| Don’t Know ☐ |

1. Has your child been admitted to Hospital for any reason other than stated previously in the last two years? If yes state what for

| Yes ☐ |
| --- |
| No ☐ |
| Don’t Know ☐ |

**DRUG HISTORY**

1. *Only answer if answered yes to having hayfever symptoms*
2. Has your child ever been prescribed any of the following for their hayfever?

| Anti-Histamines ☐ | Nasal Spray ☐ | Eye Drops ☐ |
| --- | --- | --- |
| Sublingual Immunotherapy ☐ | Subcutaneous Immunotherapy ☐ |  |
| None ☐ | Don’t know ☐ |  |

1. Is your child on any other regular prescribed medications that aren’t for Cystic Fibrosis? If so state what

**SOCIAL HISTORY**

*Thinking about the house which your child spends the most time in:*

1. Do any smokers live at the house?

| Yes – they smoke outside ☐ | Yes – they smoke inside ☐ |  | No ☐ |
| --- | --- | --- | --- |

1. Do any pets live inside the house?

| Dog ☐ | Cat ☐ | Bird ☐ |
| --- | --- | --- |
| Other: | | |
